# Bis-hydrophobic 5-(1,2-dithiolan-3-yl)pentanamide budding leads targeting brain sigma-1 receptors

**DOI:** 10.1371/journal.pone.0352906

**Published:** 2026-08-10

**Authors:** Rafael Flores, Kapembwa Musenda, Deyse Brito Barbosa, Kwan H. Cheng, Donald Sikazwe

**Affiliations:** 1 Pharmaceutical Sciences Department, Feik School of Pharmacy - University of the Incarnate Word, San Antonio, Texas, United States of America; 2 The University of Texas at Austin, Austin, Texas, United States of America; 3 Laboratory of Chemoinformatics and Biological Evaluation, Health Department, State University of Feira de Santana, Feira de Santana, Bahia, Brazil; 4 Department of Physics - Astronomy and Neuroscience Program, Trinity University, San Antonio, Texas, United States of America; University of Sahiwal, PAKISTAN

## Abstract

Multi-mechanistic sigma-1 (σ1) receptors are implicated in several neurodegenerative pathologies including Alzheimer's disease (AD). As part of an ongoing effort to create a collection of diverse, readily synthesizable, σ1 acting small molecules for anti-neurodegenerative applications, we opted to try lipoic acid (LA) derived amides. These amides are designated as “5-(1,2 dithiolan-3-yl)pentanamides” throughout this publication. Our design approach was σ1 pharmacophore based – that is, ligands possessing three key functionalities (a hydrophobic dithiolane group, a flexible amide H-bonding linker, a hydrophobic alkyl/aryl group). Twenty-one dithiolane pentanamides were therefore designed, docked, synthesized, and pharmacologically assessed for σ1/σ2 binding affinities. Compounds **2, 6, 17** possessed promising selective σ1 binding affinities (with respective Ki values of 256, 133 and 32 nM) versus the standard ligand **PD144418** (Ki = 0.08 nM). The three compounds will be used as leads in follow-up structure activity optimizations.

## 1 Introduction

**Fig 1:**
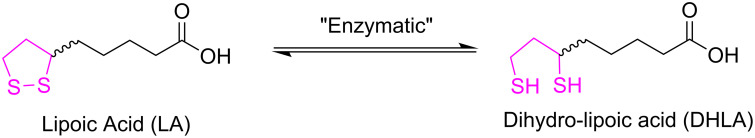
LA and DHLA redox. Physiological redox equilibration of LA and DHLA by lipoamide dehydrogenase, glutathione reductase, and thioredoxin reductase [12,16].

Gap: disease-targeted therapeutic (DTT) small molecules capable of modifying or significantly slowing AD progression are still clinically unavailable. Unabated, AD remains as an intractable progressive neurodegenerative plague that is poised to debilitate >150 million people by 2050 globally [1]. Due to the multi-factorial nature of AD, multi-mechanistic druggable targets seem to offer the opportunity to discover novel therapeutic single molecules capable of disrupting key cascades involved in disease progression. In search of central nervous system (CNS) druggable targets against AD, we decided on multi-mechanistic σ1 chaperone receptors [2]. Broadly, when σ1 receptors are activated, they bind to a diverse set of substrate proteins (ion channels included) and modify downstream signaling to yield neuronal protection in neurodegenerative disorders. In this contribution, our rationale for pursuing σ1 ligands is largely supported by such experimental evidence as: σ1 exhibits low receptor/biomarker expression densities in cognitive brain centers (prefrontal cortex, hippocampus, etc.) in early AD, σ1 agonists restored memory in AD animals and provided neuroprotection, and that σ1 activation yields anti-amnesic effects as well as stimulates synaptic plasticity [1–4]. As characterized in literature, σ1 receptors are 223 amino acid intra-mitochondria endoplasmic reticulum (ER) peptides with multi-functional signaling/neuroprotective roles [anti-inflammatory, anti-apoptosis, mitigating against reactive oxygen species (ROS) levels, increasing autophagy, β-amyloid (Aβ) plaque reduction, Ca^2+^ excitotoxicity modulation, etc.] [4–7]. To date, ligands like Blarcamesine or ANAVEX®2-73 (a σ1 agonist/M2 antagonist) have yielded promising AD clinical trials data [8,9]. Curiously, multiple central nervous system (CNS) active drugs (anti-depressants, cholinesterase inhibitors, opioids, anti-psychotics, etc.) possess σ1 activity and are being revisited for anti-neurodegenerative effects [10,11]. We therefore designed/synthesized LA based σ1 ligands to possess enhanced hydrophobic binding interactions and blood brain barrier (BBB) penetrant logP values ≥2. Our molecules feature a common dithiolane – amide motif substituted with a sampling of electronically/conformationally disparate alkyl/aryl functionalities (available in our laboratory inventory at the time, for exploratory medicinal chemistry). Secondarily, even though we were less focused on the anti-ROS aspects of the derivatives, LA’s dithiolane antioxidant properties are worth mentioning because this can be a synergistically beneficial mechanism. Essentially, under physiological conditions the dithiolane moiety equilibrates as oxidized/cyclized LA and reduced/open ring metabolite dihydrolipoic acid (DHLA, Fig 1), and both molecules are known direct/indirect antioxidants [12]. It is further indicated that the antioxidant/anti-inflammatory properties partly reside in LA’s chelation of Zn, Pb, and Cu plus the more robust DHLA’s complexation with Fe, Zn, Hg, Pb, and Cu [13,14]. Interestingly, the metal hypothesis of AD implicates Al, Fe, Zn, and Cu imbalances as contributory factors [15].

**Fig 2:**
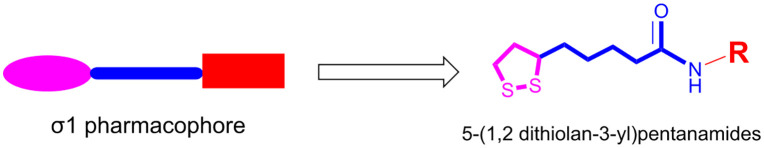
Sigma 1 ligands pharmacophore. The σ1 “hydrophobic/H-bond linker/hydrophobic” pharmacophore model represented by the dithiolane/flexible amide linker/alkyl or aryl R-substituents in the target compounds. The flexible amide linker, which is amenable to chain length variation and conformational constraint, will be explored in future structure activity relationship (SAR) studies of our three leads.

This manuscript shares aspects of design, synthesis, and σ1 pharmacological binding affinities of twenty-one hydrophobic 5-(1,2-dithiolan-3-yl)pentanamides. Design considerations included structural adherence to the reported σ1 ligand’s pharmacophore illustrated in Fig 2 [17]. This pharmacophore proposes that σ1 ligand’s chemical basis of action is related to their ability to form a minimum of two hydrophobic and one H-bonding interactions within receptor’s active site [17].

Additionally, all target compounds were computationally evaluated for σ1 receptor binding affinity, active site interactions, and drug-likeness [per *Lipinski’s rule of five”* or RoF, plus absorption/distribution/metabolism/elimination (ADME) attributes], prior to synthesis [18,19]. All synthesized compound structures were confirmed using proton (^1^H) and carbon (^13^C) nuclear magnetic resonance (NMR) experiments, and high-resolution mass spectrometry (HR-MS) analysis. In vitro σ1/σ2 receptor binding affinities are also determined for all compounds.

## 2 Results and Discussion

### 2.1 Synthesis

**Fig 3:**
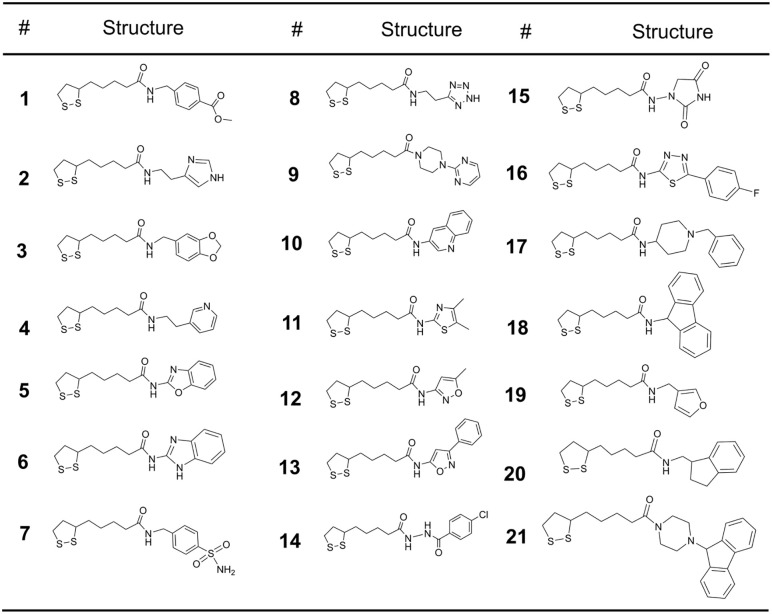
Compound structures. Twenty-one target compounds CAS SciFinder^®^ searches revealed that compounds **5, 8-13, 15, 16, 18-21** are new, while **1-4**, **6, 7, 14, 17** are known and reported elsewhere for disparate applications [22–29]. Componds **2**, **6**, **17** exhibited promising selective *in vitro* binding affinities at σ1.

**Scheme 1:**
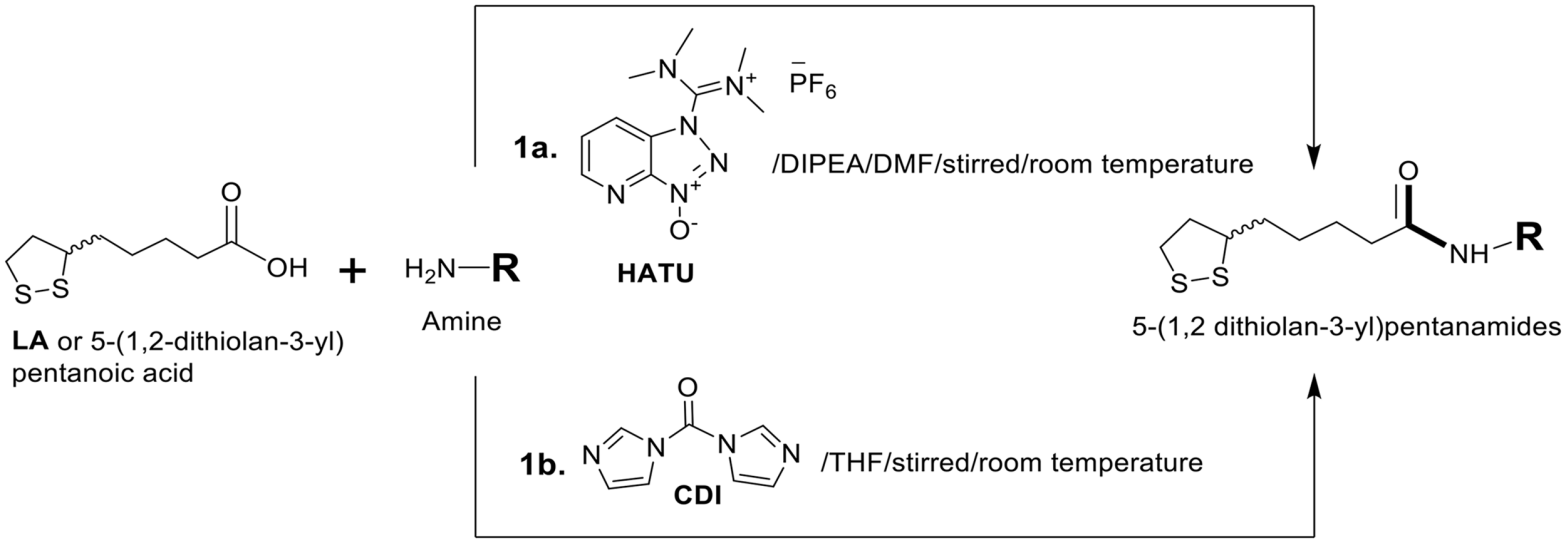
One-step amidation using either HATU (1a, diisopropylethylamine or DIPEA in dimethylformamide or DMF) or CDI (1b, in tetrahydrofuran or THF) coupling conditions.

Designed/modelled pentanamides were conveniently synthesized in parallel, using slightly modified one-pot amidation conditions. Synthesis was achieved through direct coupling reactions between LA [also called “5-(1,2-dithiolan-3-yl)pentanoic acid”] and appropriate alkyl-/aryl-amines, aided by either “1-[bis(dimethylamino)methylene]-1H-1,2,3-triazolo[4,5-b]pyridinium 3-oxide hexafluorophosphate (HATU)” *or “*1,1’-carbonyldiimidazole (CDI)” (Scheme 1) [20,21]. The ensuing twenty-one target compounds are indicated in Fig 3.

### 2.2 Docking simulations

The protein data bank (PDB) yielded σ1 receptor 3D structure (i.e., PDB ID: 5HK1 plus crystallographic ligand: PD144418) [30]. Removal of H_2_O and crystallization artifacts from the 3D structure were achieved with Pymol and Gasteiger-Huckel, charges were added together with hydrogen atoms to optimize their hydrogen bonds [31]. Conformational searches and scoring were performed based on the crystallographic ligand interaction locations. Scoring functions were obtained via the Autodock Vina 1.1.2 platform [32]. The search area was delineated by Autodock Tools 1.5.7 program, by defining a box with x = 12 Å; y = 12 Å; and z = 16 Å dimensions [33]. Table 1 indicates the predicted affinity scores (kcal/mol) for our compounds versus the high affinity reference σ1 ligand **PD144418**. Lower or more negative free energy (kcal/mol) scores suggest higher binding affinities.

**Table 1:** Autodock Vina binding scores (kcal/mol) for compounds 1 - 21 and PD144418. *In vitro* promising molecules (2, 6, 17) exhibited low to comparable predicted affinity scores versus PD144418.

Compounds	Score (kcal/mol)	Compounds	Score (kcal/mol)
1	−8.7	12	−7.9
**2**	**−7.4**	13	−10.3
3	−9.1	14	−9.5
4	−8	15	−8.1
5	−8.9	16	−9.5
**6**	**−9**	**17**	**−9.7**
7	−8.6	18	−8.9
8	−7.7	19	−7.7
9	−9	20	−9.8
10	−9.7	21	−9.7
11	−8	**PD144418**	**−9.9**

**Fig 4:**
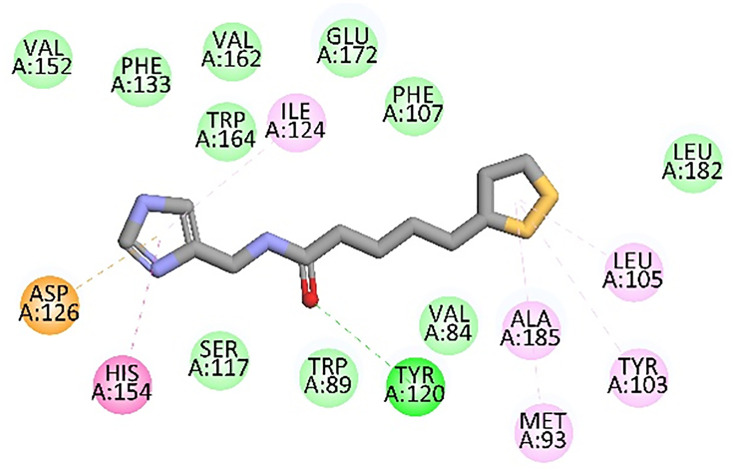
Compound 2’s interactions map. Bonding interaction map of **2** at σ1 active site. Interactions were colored using the following scheme: van der Waals (light green); conventional hydrogen bonds (bright green); π-donor or carbon hydrogen bonds (pale green); π-anion/attractive charge interactions (orange); π-sigma (purple); π-sulfur (yellow); π-π stacked and π-π T-shaped interactions (magenta); and alkyl/π-alkyl interactions (light pink).

**Fig 5:**
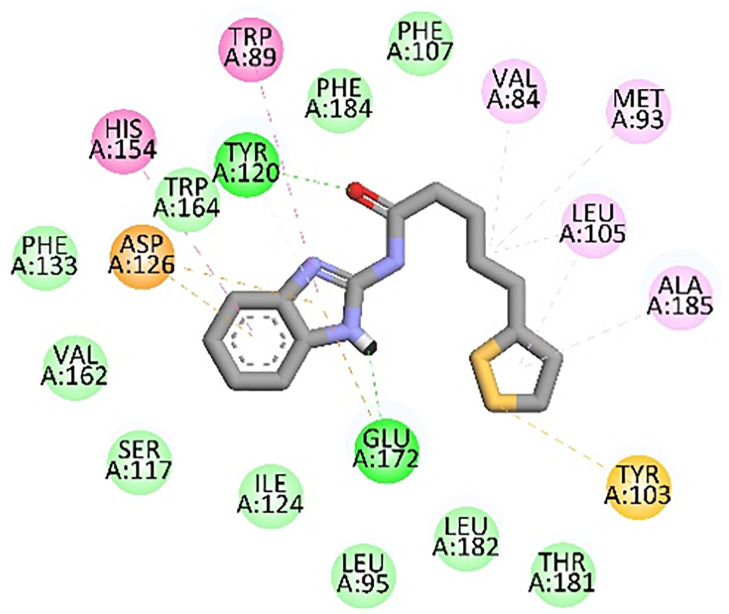
Compound 6’s interactions map. Interaction map of compound **6** at σ1 active site. The interaction color scheme is as described for Fig 4.

**Fig 6:**
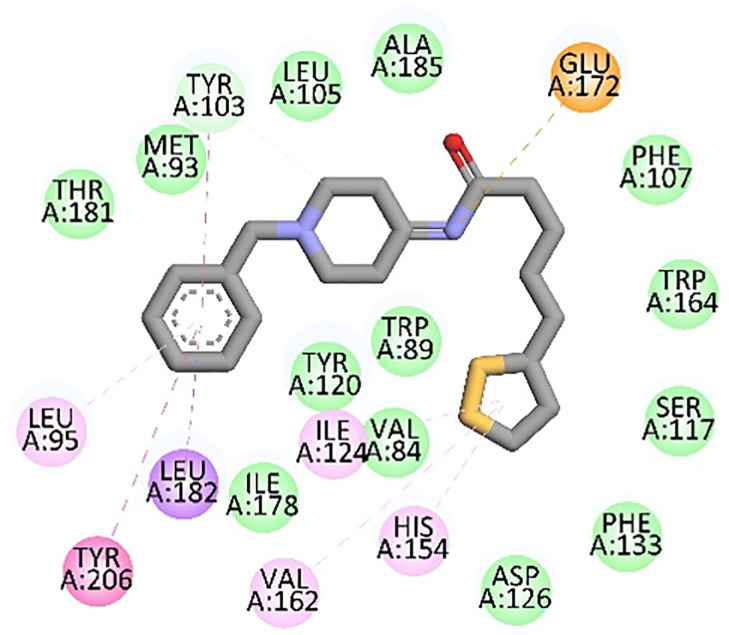
Compound 17’s interactions map. Interaction map of **17** at σ1 active site. Fig 4 interaction color scheme applies here as well.

Interaction maps (Figs 4–6) of the three promising leads (compounds **2**, **6**, **17**) were generated using Discovery Studio Visualizer [34].

Compound **2** (predicted affinity score = −7.4 kcal/mol) aromatic imidazole establishes three different pi-bonds (π-bonds): π-anion with Asp126, π-stacking with His154 and π-alkyl with Ile124. The carbonyl group hydrogen bonds (H-bonds) with Tyr120, and the dithiolane ring alkylates with Met93, Tyr103, Leu105, and Ala185. Additionally, several hydrophobic interactions can be established along the ligand extension, with residues as Val84, Trp89, Phe107, Ser117, Phe133, Val152, Val162, Trp164, Glu172 and Leu182.

Molecule 6 (score = −9.0 kcal/mol) bonds to σ1 via dithiolane alkyl/Leu105/Ala185 and π-sulfur interaction with Tyr103. Additionally, the alkyl linker establishes alkyl interactions with Val84, Met93, and Leu105. H-bonds occur between the carbonyl oxygen and Tyr120, and between the imidazole amine and Glu172. The benzimidazole group π-bonds to Trp89, Asp126, His154, and Glu172. Hydrophobic interactions are observed with residues Leu95, Phe107, Ser117, Ile124, Phe133, Val162, Trp164, Thr181, Leu182, and Phe184.

High affinity compound **17** (score = −9.7 kcal/mol) establishes benzyl ring π-bonds with Leu95, Tyr103, Leu182, and Tyr206. The dithiolane group forms alkyl interactions with Ile124, His154 and Val162, while the charged nitrogen interacts with Glu172. Val84, Trp89, Tyr120, Met93, Tyr103, Leu105, Phe107, Ser117, Asp126, Phe133, Trp164, Ile178, Thr181, and Ala185 participate in hydrophobic bonding.

Overall, prior studies involving high-affinity inhibitors described residues Tyr103 and Glu172 as being fundamental to σ1 inhibition [30]. In this study, ligands capable of establishing stronger interactions with these residues also exhibited more favorable predicted binding affinity scores. Our interaction profile suggested that compounds capable of forming electrostatic interactions with Glu172 and H-bonds with Tyr103 exhibited more favorable binding energies. Conversely, ligands interacting mainly through van der Waals contacts with the same residues showed comparatively weaker predicted affinities. The interaction maps and affinity scores also suggested a correlation between *in vitro* activity and predicted binding affinities, meaning that increased biological activity was related to lower docking scores.

### 2.3 Drug likeness

Drug likeness molecular filters are plentiful, and they all attempt to indicate the prevalence of substructures among marketed drug molecules [35]. Lipinski’s RoF oral drug-like filters are mostly related to the compound’s molecular weight (MW), lipophilicity (i.e., calculated log octanol/water coefficient or ClogP values), H-bond donors (HBDs) and H-bond acceptors (HBAs) [17,36]. Alternatively, the multiparameter optimization (MPO) algorithm considers MW, ClogP, calculated log distribution (ClogD, pH = 7.4) coefficient, topological polar surface area (TPSA), HBDs, and pKa as drug likeness filters for CNS molecules [37]. Moreover, H_2_O solubility (log*S*) is also an important parameter, it influencesdrug absorption and distribution…low H_2_O solubility implies variable to poor drug oral bioavalability.We deployed the open-source SwissADME pharmacokinetic tool to predict some of the above indicated attributes plus Cyp450 enzyme disposition early in drug design, prior to undertaking any chemical synthesis [19]. The main reasons for using SwissADME were ease of accessibility and multi-parametric robustness. Illustrated in Table 2 are molecular weight (MW), consensus ClogP values, TPSA, and drug-likeness (Dl) scores of our target compounds. Complete SwissADME predicted pharmacokinetic properties for the individual compounds are listed in S1 File.

**Table 2:** This table displays compound MWs and sample SwissADME generated pharmacokinetic scores including “drug-likeness.”.

Compounds	MW	HBDs	HBAs	ClogP_*o/w*_	TPSA	Dl
**1**	353.5	1	3	3.33	106	Yes
**2**	299.46	2	2	2.24	108.38	Yes
**3**	339.47	1	3	3.23	98.16	Yes
**4**	310.48	1	2	2.88	92.59	Yes
**5**	322.45	1	3	3.29	105.73	Yes
**6**	321.46	2	2	3.1	108.38	Yes
**7**	374.54	2	4	2.22	148.24	Yes
**8**	316.51	1	2	3.51	120.83	Yes
**9**	301.43	2	4	1.59	134.16	Yes
**10**	352.52	0	3	2.22	99.93	Yes
**11**	332.48	1	2	3.72	92.59	Yes
**12**	286.41	1	3	2.49	105.73	Yes
**13**	348.48	1	3	3.64	105.73	Yes
**14**	358.91	2	2	3.27	108.8	Yes
**15**	303.4	2	3	0.9	129.11	Yes
**16**	383.53	1	4	4.16	133.72	Yes
**17**	378.6	1	2	3.75	82.94	Yes
**18**	369.54	1	1	4.52	79.7	Yes
**19**	285.43	1	2	2.69	92.84	Yes
**20**	335.53	1	1	4.01	79.7	Yes
**21**	438.65	0	2	4.47	74.15	Yes

Our compounds met the Lipinski RoF guidelines [MWs < 500, HBDs (NH/OH ≤ 5 moieties) and HBAs (O/N ≤ 10 atoms), calculated logPs < 5, etc.]. Ostensibly, lipophilic small MW molecules have enhanced chances of traversing endothelial cells of the blood brain barrier (BBB) [38]. Apart from **15** (ClogP = 0.9), the rest of the molecules possessed moderate to highly lipophilic (ClogP values 1.6–4.5). In fact, compound **17**, with a CLogP = 3.75 and 83Å^2^ TPSA, has good brain penetrant potential. Reportedly, TPSA ≤ 90Å^2^ scores imply enhanced brain bioavailability [39]. According to the “organic chemistry portal (https://www.organic-chemistry.org/prog/peo/logS.html),” most of our compounds fell in the *very* to *moderate* H_2_O solubility range of most marketed drugs (SwissADME S1 File).

### 2.4 Receptor binding affinities

Compound affinity experiments (σ1/σ2 K_i_ values) were conducted by the “National Institute of Mental Health’s Psychoactive Drug Screening Program (NIMH PDSP)”, at University of North Carolina (UNC) Chapel Hill, NC. Protocol details of the competitive radioligand displacement assays utilized are reported elsewhere [40]. Table 3 below indicates the K_i_ values for all compounds.

**Table 3:** The binding affinity (Ki) data set at both σ1 and σ2. Molecules 2, 6, 17 exhibited the best and selective saturation radioligand affinities for σ1. Notably, PD144418 binding affinity for σ1 is exceptionally high hence its use as a comparator ligand [41]. ND = Not Determined (>10,000 nM activity).

Compounds	σ-1 Ki (nM)	σ-2 Ki (nM)
**1**	ND	ND
**2**	**255.65**	**ND**
**3**	ND	ND
**4**	>1000	ND
**5**	ND	ND
**6**	**133.12**	**ND**
**7**	**ND**	ND
**8**	>1000	ND
**9**	ND	ND
**10**	ND	ND
**11**	ND	ND
**12**	ND	ND
**13**	ND	ND
**14**	ND	ND
**15**	ND	ND
**16**	ND	ND
**17**	**31.73**	**523.45**
**18**	ND	ND
**19**	ND	ND
**20**	ND	ND
**21**	ND	ND
**PD 144418**	**0.08**	**>1000**

## 3 Conclusion

Twenty-one derivatives were successfully designed based on the “hydrophobic pharmacophore” concept, modelled (via affinity scores, for active site interactions, and for ADME), synthesized, and characterized (by NMR and HRMS). Derivatives were tested *in vitro* for binding affinities at σ1/σ2. Compounds **2, 6, 17** structures were well accommodated within σ1 active site and established multiple van der Walls interactions with residues around the gorge – an indicator of stable binding. SwissADME calculations indicated that the derivatives were lipophilic and drug-like. Additionally, **2, 6, 17** exhibited respective selective *in vitro* affinities of 255, 133, 31 nM (Ki values) at σ1. Thusly, the three compounds are promising *leads* for future structure optimization research on LA inspired hydrophobic σ1 ligands.

## 4 Experimental

### 4.1 General

Synthetic chemicals were sourced from several vendors (Thermo Fisher Scientific, Millipore Sigma, etc.) and used in the purity (>95%) received. Melting points were obtained using the “Mel-temp” instrumentand reported as uncorrected values. Product purifications were conducted using the “Teledyne Combiflash Rf” flash chromatography. “Silica Gel 60 F_254_ TLC plates” were utilized for monitoring reaction progress using either the hexanes/ethylacetate (7:3) or dichloromethane/methanol (9:1) co-solvents. Compound spectra (^1^H/^13^C) were obtained using Bruker 300 MHz NMR spectroscopy (deuterated DMSO-d_6_ or CDCl_3_ solvents). Chemical shifts ^1^H/^13^C are reported versus Tetramethylsilane (TMS or SiMe_4_) internal standard. Structural data are reported as “chemical shift (ppm), multiplicity (s = singlet, d = doublet, t = triplet, q = quartet, br = broad, m = multiplet), coupling constants (*J* in Hz), proton numbers, and mass to charge ratio” (S2 File). HR-MS data were collected on a “maXis plus quadrupole-time of flight mass spectrometer equipped with an electrospray ionization source (Bruker Daltonics),” in positive ionization mode (S3 File). HR-MS experiments were conducted by the Univ. of Texas at San Antonio (UTSA) Mass Spectrometry & Proteomics Core Facility.

### 4.2 Chemistry

**HATU coupling procedure (A)**. A mixture of (*R/S*)-lipoic acid (1 eq), HATU (1.2 eq), the appropriate alkyl-/aryl-amine (1.2 eq), dimethylformamide (DMF) was stirred in a round bottom (RB) flask, under N_2_. Diisopropylethylamine (DIPEA, 2.2 eq) was then injected dropwise and reaction progress was monitored by thin layer chromatography (TLC). After 24 hours, the reaction mixture was diluted with 15 mL of dichloromethane (DCM), successively washed with deionized water (3 X 20mL) and extracted with DCM (3 X 20mL). Organic extracts were combined, dried over Na_2_SO_4_ and concentrated under vacuum. The crude product was purified by flash chromatography (Combi-Flash Rf) using either ethyl acetate using hexane/ethyl acetate (7:3) or DCM/MeOH (9:1) gradient solvent systems.

**CDI coupling procedure (B)**. The appropriate alkyl-/aryl-amine (1.1 eq) was added in one portion to a cooled RB flask charged with (*R/S*)-lipoic acid (1 eq), 1,1’-Carbonyldiimidazole (CDI, 1.2 eq), in tetrahydrofuran (THF), under N_2_, and stirred. Reaction progress was monitored over 24 hours by TLC. Solvent was evaporated, and the oily product mixture was diluted with water (15 mL) then extracted with DCM (3 X 15 mL). Combined organic phases of the crude product were dried over Na_2_SO_4_ and purified by Combi-Flash Rf, using either ethyl acetate using either hexane/ethyl acetate (7:3) or DCM/MeOH (9:1) as gradient eluent solvents.

### 4.3 Synthetic specifics and spectral data

**[Methyl 4-((5-(1,2-dithiolan-3-yl)pentanamido)methyl)benzoate]** (**1)** [22]**:** (*R*/*S*)-lipoic acid (*R/S*-LA) (500 mg, 2.4 mmol) was reacted with methyl 4-(aminomethyl) benzoate hydrochloride (587 mg, 2.9 mmol) and DIPEA (870 µL, 5 mmol) in DMF (10 mL) at room temperature for 24 h with HATU (1.1 g, 2.9 mmol) as the coupling reagent. The desired product was isolated, using procedure “A”, as a yellowish crystalline solid. (658 mg), yield: 77%; mp: 100−102 ℃; ^1^HNMR(300 MHz, DMSO-d_6_): δ 8.44 (t, J = 5.96 Hz 1H, NH), δ 7.92 (d, J = 8.31 Hz, 2H, CH Ar), δ 7.37 (d, J = 8.32 2H, CH Ar), δ 4.33 (d, J = 5.96, 2H, CH_2_), δ 3.84 (s, 3H, CH_3_), δ 3.67–3.55 (m, 1H, CH_2_), δ 3.24–3.06 (m, 2H, CH_2_), δ 2.46–2.34 (m, 1H, CH), δ 2.17 (t, J = 7.20 Hz, 2H, CH_2_), δ 1.93–1.79 (m, 1H, CH) δ 1.74–1.47 (m, 4H, 2CH_2_), δ 1.43–1.28 (m, 2H, CH_2_); ^13^CNMR (300 MHz, CDCl_3_): 172.6670, 166.8073, 143.5963, 130.0277, 129.3613, 127.5786, 56.4007, 52.1523, 43.2173, 40.2500, 38.4862, 36.4009, 34.6082, 28.8859, 25.3570; HRMS (ESI) m/z calcd. for C_17_H_23_NO_3_S_2_ [(M + H)^+^] 354.1192, found 354.1190.

**[N-(2-(1H-imidazol-4-yl)ethyl)-5-(1,2-dithiolan-3-yl)pentanamide] (2)** [23,30]**:** *R/S*-LA (309 mg, 1.5 mmol) was reacted histamine dihydrochloride (326 mg, 1.8 mmol) and DIPEA (1mL, 5.4 mmol) in DMF (5 mL) at room temperature for 24 h with HATU (698 mg, 1.8 mmol) as the coupling reagent. The desired product was isolated, using procedure “A”, as a yellowish crystalline solid. (170 mg), yield: 24%; mp: 99−101 ℃; ^1^HNMR (300 MHz, DMSO-d_6_): δ 11.78 (s, 1H, NH), δ 7.85 (s, 1H, NH), δ 7.51 (s, 1H, CH Ar), δ 6.84–6.65 (m, 1H, CH Ar), δ 3.60 (m, 1H, CH), δ 3.30–3.07 (m, 4H, 2CH_2_), δ 2.75–2.54 (m, 2H, CH_2_), δ 2.48–2.35 (m, 1H, CH), δ 2.05 (t, J = 7.24 Hz, 2H, CH_2_), δ 1.93–1.80 (m, 1H, CH), δ 1.74–1.44 (m, 4H, 2CH_2_), δ 1.42–1.25 (m, 2H, CH_2_); ^13^CNMR(300 MHz, DMSO-d_6_): 172.2558, 135.0466, 56.5985, 38.5690, 35.7060, 34.5980, 28.7620, 27.5202, 25.5169; HRMS (ESI) m/z calcd. for C_13_H_21_N_3_OS_2_ [(M + H)^+^] 300.1199, found 300.1196.

**[N-(benzo[d] [1,3]dioxol-5-ylmethyl)-5-(1,2-dithiolan-3-yl)pentanamide] (3)** [24]: *R/S*-LA (500 mg, 2.4 mmol) was reacted with piperonylamine (440 mg, 2.9 mmol) and DIPEA (1.3 mL, 7 mmol) in THF (10 mL) at room temperature for 24 h with CDI (470 mg, 2.9 mmol) as the coupling reagent. The desired product was isolated, using procedure “B”, as a yellowish crystalline solid. (362 mg), yield: 44%; mp: 84−87 ℃; ^1^HNMR (300 MHz, DMSO-d_6_): δ 8.24 (t, 1H, NH), δ 6.83 (t, 2H, 2CH Ar), δ 6.71 (m, 1H, CH Ar), δ 5.98 (s, 2H, CH_2_), δ 4.15 (d, J = 5.93 Hz, 2H, CH_2_), δ 6.37–3.54 (m, 1H, CH), δ 3.24–3.05 (m, 2H, CH_2_), δ 2.47–2.34 (m, 1H, CH), δ 2.12 (t, J = 7.29 Hz, 2H, CH_2_), δ 1.92–1.78 (m, 1H, CH), δ1.74–1.46 (m, 4H, 2CH_2_), δ 1.43–1.23 (m, 2H, CH_2_); ^13^CNMR(300 MHz, DMSO-d_6_): 172.3171, 147.6776, 146.4375, 134.1199, 120.8179, 108.4243, 108.3237, 101.2513, 56.5913, 42.2392, 38.5715, 35.6051, 34.5649, 28.7664, 25.5120; HRMS (ESI) m/z calcd. for C_16_H_21_NO_3_S_2_ [(M + H)^+^] 340.1036, found 340.1032.

**[5-(1,2-dithiolan-3-yl)-N-(2-(pyridin-3-yl)ethyl)pentanamide] (4)** [25]**:** *R/S*-LA (500 mg, 2.4 mmol) was reacted with 3-(2-aminoethyl)pyridine (350 µL, 2.9 mmol) and DIPEA (840 µL, 4.6 mmol) in THF (10 mL) at room temperature for 24 h with CDI (470 mg, 2.9 mmol) as the coupling reagent. The desired product was isolated, using procedure “B”, as a yellowish crystalline solid. (380 mg), yield: 50%; mp: 53−56 ℃; ^1^HNMR (300 MHz, DMSO-d_6_): δ 8.42 (m, 2H, CH Ar), δ 7.89 (m, 1H, NH), δ 7.62 (m, 1H, CH Ar), δ 7.35–7.27 (m, 1H, CH Ar), δ 3.65–3.53 (m, H, CH), δ 3.34–3.07 (m, 4H, CH_2_), δ 2.72 (t, J = 6.97 Hz, 2H, CH_2_), 2.47–2.35 (m, 1H, CH), δ 2.03 (t, J = 7.29 Hz, 2H, CH_2_), δ 1.93–1.78 (m, 1H, CH), δ 1.72–1.40 (m, 4H, 2CH_2_), δ 1.38–1.20 (m, 2H, CH_2_); ^13^CNMR(300 MHz, DMSO-d_6_): 172.3717, 150.3380, 147.8308, 136.6230, 135.4389, 123.8265, 56.5837, 38.5767, 35.6485, 34.5931, 32.6615, 28.7346, 25.497; HRMS (ESI) m/z calcd. for C_15_H_22_N_2_OS_2_ [(M + H)^+^] 311.1246, found 311.1243.

**[N-(benzo[d]oxazol-2-yl)-5-(1,2-dithiolan-3-yl)pentanamide] (5):** *R/S*-LA (500 mg, 2.4 mmol) was reacted with 2-Aminobenzoxazole (390 mg, 2.9 mmol) and DIPEA (840 µL, 4.6 mmol) in DMF (10 mL) at room temperature for 24 h. HATU (1.1g, 2.9 mmol) was the coupling reagent. The desired product was isolated, using procedure “A”, as a yellowish crystalline solid. (251 mg), yield: 32%; mp: 109−111 ℃; ^1^HNMR(300 MHz, DMSO -d_6_): δ 11.60 (s, 1H, NH), δ 7.64–7.54 (m, 2H, 2CH Ar), δ 7.36–7.21 (m, 2H, 2CH Ar), δ 3.71–3.58 (m, 1H, CH), δ 3.26–3.04 (m, 2H, CH_2_), δ 2.57–2.35 (m, 3H, CH_2_ CH), δ 1.97–1.81 (m, 1H, CH), δ 1.79–1.52 (m, 4H, 2CH_2_) δ 1.50–1.34 (m, 2H, CH_2_); ^13^CNMR(300 MHz, DMSO-d_6_): 171.2725, 155.5937, 148.0528, 141.1830, 124.9585, 123.9179, 118.6209, 110.4518, 56.5256, 40.3799, 38.5954, 36.1424, 34.5871, 28.6218, 24.6285; HRMS (ESI) m/z calcd. for C_15_H_18_N_2_O_2_S_2_ [(M + H)^+^] 323.0882, found 323.0877.

**[N-(1H-benzo[d]imidazol-2-yl)-5-(1,2-dithiolan-3-yl)pentanamide] (6)** [26]**:** *R/S*-LA (500 mg, 2.4 mmol) was reacted with 2-Aminobenzoxazole (400 mg, 2.9 mmol) and DIPEA (1 mL, 5.4 mmol) in DMF (10 mL) at room temperature for 24 h with HATU (1.1g, 2.9 mmol) as the coupling reagent. The desired product was isolated, using procedure “A”, as a yellowish crystalline solid (350 mg), yield: 45%; mp: 217−219 ℃; ^1^HNMR(300 MHz, DMSO-d_6_): δ 12.03 (s, 1H, NH), δ 11.48 (s, 1H, NH), δ 7.43 (s, 2H, 2CH Ar), δ 7.12–7.02 (m, 2H, 2CH Ar), δ 3.71–3.58 (m, 1H, CH), δ 3.26–3.07 (m, 2H, CH_2_), δ 2.50–2.35 (m, 3H,CH CH_2_), δ 1.96–1.81 (m, 1H, CH), δ 1.80–1.52 (m, 4H, 2CH_2_), δ 1.50–1.35 (m, 2H, CH_2_); ^13^CNMR(300 MHz, DMSO-d_6_): 172.8391, 147.0740, 121.3321, 56.5259, 40.3861, 38.5882, 35.7100, 34.5913, 28.6689, 24.9987, HRMS (ESI) m/z calcd. for C_15_H_19_N_3_OS_2_ [(M + H)^+^] 322.1042, found 322.1037.

**[5-(1,2-dithiolan-3-yl)-N-(4-sulfamoylbenzyl)pentanamide] (7)** [27]**:** *R/S*-LA (500 mg, 2.4 mmol) was reacted with 4-(Aminomethyl)benzenesulfonamide hydrochloride (643 mg, 2.9 mmol) and DIPEA (1 mL, 5.4 mmol) in DMF (10 mL) at room temperature for 24 h with HATU (1.1g, 2.9 mmol) as the coupling reagent. The desired product was isolated, using procedure “A”, as a yellowish solid (580 mg), yield: 64%; mp: 114−117 ℃; ^1^HNMR(300 MHz, DMSO-d_6_): δ 8.42 (t, J = 5.87 Hz, 1H, NH), δ 7.77 (d, J = 8.25 Hz, 2H, 2CH Ar), δ 7.41 (t, J = 8.34 Hz,2H, 2CH), δ 7.32 (s, 2H, NH_2_), δ 4.32 (d, J = 5.96 Hz, 2H, CH_2_), δ 3.68–3.55 (m, 1H, CH), δ 3.25–3.07(m, 2H, CH_2_), δ 2.47–2.35 (m, 1H, CH), δ 2.16 (t, J = 7.29 Hz, 2H, CH_2_), δ 1.93–1.80 (m, 1H, CH), δ 1.76–1.48 (m, 4H, 2CH_2_), δ 1.45–1.29 (m, 2H, CH_2_); ^13^CNMR(300 MHz, DMSO-d_6_): 172.6110, 144.3358, 143.0168, 127.9082, 126.1365, 56.5922,42.1196, 40.3884, 38.5944, 35.5842, 34.5887, 28.7991, 25.4739; HRMS (ESI) m/z calcd. for C_15_H_22_N_2_O_3_S_3_ [(M + H)^+^] 375.0865, found 375.0858.

**[N-(4,5-dimethylthiazol-2-yl)-5-(1,2-dithiolan-3-yl)pentanamide] (8):** *R/S*-LA (500 mg, 2.4 mmol) was reacted with 2-Amino-4,5-dimethylthiazole hydrochloride (440 mg, 2.7 mmol) and DIPEA (600 µL, 3.6 mmol) in DMF (10 mL) at room temperature for 24 h with HATU (1.1g, 2.9 mmol) as the coupling reagent. The desired product was isolated, using procedure “A”, as a yellowish solid (133 mg), yield: 25%; mp: 108−110 ℃; ^1^HNMR(300 MHz, CDCl_3_): δ 10.70 (s, 1H, NH), δ 3.63–3.48 (m, 1H, CH), δ 3.25–3.05 (m, 2H, CH_2_), δ 2.53–2.37 (m, 3H, CH CH_2_), δ 2.31 (s, 3H, CH_3_), δ 2.25 (s, 3H, CH_3_), δ 1.97–1.82 (m, 1H, CH), δ 1.82–1.60 (m, 4H, 2CH_2_), δ 1.59–1.38 (m, 2H, CH_2_), ^13^CNMR, *δ* (ppm) (300 MHz, DMSO-d_6_): 171.0700, 154.0563, 141.9496, 118.6859, 56.4994, 40.3719, 38.5884, 35.1804, 34.5280, 28.6640, 24.9946, 14.7479, 10.7797; HRMS (ESI) m/z calcd. for C_13_H_20_N_2_OS_3_ [(M + H)^+^] 317.0811, found 317.0807.

**[N-(2-(2H-tetrazol-5-yl)ethyl)-5-(1,2-dithiolan-3-yl)pentanamide] (9):** *R/S*-LA (500 mg, 2.4 mmol) was reacted with 2-(2H-Tetrazol-5-yl)ethan-1-amine hydrochloride (407 mg, 2.7 mmol) and DIPEA (600 µL, 3.6 mmol) in DMF (10 mL) at room temperature for 24 h with HATU (1.1g, 2.9 mmol) as the coupling reagent. The desired product was isolated, using procedure “A”, as a yellowish solid (160 mg), yield: 22%; mp: 141−143 ℃; ^1^HNMR(300 MHz, DMSO-d_6_): δ 7.98 (m, 1H, NH), δ 3.67–3.54 (m, 1H, CH), 3.46–3.36 (m, 1H, CH), 3.25–3.08 (m, 3H, CH CH_2_), δ 3.01 (t, J = 6.92 Hz, 2H, CH_2_), δ 2.48–2.34 (m, 1H, CH), δ 2.03 (t, J = 7.29 Hz, 2H, CH_2_), δ 1.92–1.79 (m, 1H, CH), δ 1.71–1.41 (m, 4H, 2CH_2_), δ 1.38–1.23 (m, 2H, CH_2_); ^13^CNMR(300 MHz, DMSO-d_6_): 172.6897, 154.5424, 56.5489, 40.3558, 38.5706, 37.1641, 35.5487, 34.5708, 28.6870, 25.3849, 23.9667; HRMS (ESI) m/z calcd. for C_11_H_19_N_5_OS_2_ [(M + H)^+^] 302.1104, found 302.1103.

**[5-(1,2-dithiolan-3-yl)-1-(4-(pyrimidin-2-yl)piperazin-1-yl)pentan-1-one] (10):** *R/S*-LA (352 mg, 1.7 mmol) was reacted with 2-(1-Piperazinyl)pyrimidine (306 mg, 1.9 mmol) in THF (5 mL) at room temperature for 24 h with CDI (423 mg, 2.6 mmol) as the coupling reagent. The desired product was isolated, using procedure “B”, as a yellowish solid (123 mg), yield: 15%; mp: 50−53 ℃; ^1^HNMR(300 MHz, CDCl_3_): δ 8.26 (d, J = 4.77 Hz 2H, 2CH Ar), δ 6.48 (d, J = 4.72 Hz, 1H, CH Ar), δ 3.85–3.70 (m, 4H, 2CH_2_), δ 3.69–3.59 (m, 2H, CH_2_), δ 3.58–3.43 (d, 3H, CH CH_2_), δ 3.18–3.00 (m, 2H, CH_2_), δ 2.47–2.27 (m, 3H, CH CH_2_), δ 1.93–1.79 (m, 1H, CH), δ 1.75–1.36 (d, 6H, 3CH_2_); ^13^CNMR(300 MHz, CDCl_3_): 171.4495, 161.4944, 157.7829, 110.4687, 56.4354, 45.3397, 43.7958, 43.5694, 41.3734, 40.2574, 38.5135, 34.7812, 33.0970, 29.1107, 24.9619; HRMS (ESI) m/z calcd. for C_16_H_24_N_4_OS_2_ [(M + H)^+^] 353.1464, found 353.1457.

**[5-(1,2-dithiolan-3-yl)-N-(quinolin-3-yl)pentanamide] (11):** *R/S*-LA (500 mg, 2.4 mmol) was reacted with 3-Aminoquinoline (419 mg, 2.9 mmol) and DIPEA (800 µL, 5 mmol) in DMF (6 mL) at room temperature for 24 h with HATU (1.1g, 2.9 mmol) as the coupling reagent. The desired product was isolated, using procedure “A”, as a yellowish solid (168 mg), yield: 21%; mp: 119−121 ℃; ^1^HNMR (300 MHz, CDCl_3_): δ 8.78 (s, 2H, 2CH Ar), δ 8.23 (s, 1H, NH), δ 8.02 (d, J = 8.34 Hz, 1H, CH Ar), δ 7.78 (d, J = 8.16 Hz 2H, 2CH Ar), δ 7.63 (m, 1H, CH Ar), δ 7.53 (m, 1H, CH Ar), δ 3.63–3.50 (s, 1H, CH), δ 3.24–3.04 (m, 2H, CH_2_), δ 2.54–2.37 (m, 3H, CH CH_2_), δ 1.96–1.41 (s, 7H, 3CH_2_ CH); ^13^CNMR(300 MHz, CDCl_3_): 171.9492, 145.0323, 143.8637, 131.6763, 128.7711, 128.4013, 128.2611, 127.8164, 127.3673, 124.0907, 56.3991, 40.2752, 38.5120, 37.2851, 34.6210, 28.8753, 25.1630, HRMS (ESI) m/z calcd. for C_17_H_20_N_2_OS_2_ [(M + H)^+^] 333.1090, found 333.1085.

**[5-(1,2-dithiolan-3-yl)-N-(5-methylisoxazol-3-yl)pentanamide] (12):** *R/S*-LA (500 mg, 2.4 mmol) was reacted with 3-amino-5-methylisoxazole (300 mg, 2.9 mmol) and DIPEA (800 µL, 5 mmol) in DMF (10 mL) at room temperature for 24 h with HATU (1.1 g, 2.9 mmol) as the coupling reagent. The desired product was isolated, using procedure “B”, as a yellowish solid (214 mg), yield: 31%; mp: 88−90 ℃; ^1^HNMR(300 MHz, CDCl_3_): δ 9.88 (s, 1H, CH Ar), δ 6.70 (s, 1H, CH Ar), δ 3.58–3.45 (m, 1H, CH), δ 3.17–2.99 (m, 2H, CH_2_), δ 2.48–2.37 (m, 3H, CH CH_2_), δ 2.35 (s, 3H, CH_3_), δ 1.91–1.77 (m, 1H, CH), δ 1.76–1.58 (s, 4H, 2CH_2_), δ 1.57–1.35 (s, 2H, CH_2_); ^13^CNMR(300 MHz, CDCl_3_): 171.4389, 169.9553, 158.4442, 96.6661, 56.3522, 40.2336, 38.5153, 36.7670, 34.6425, 28.8276, 24.9810,12.7245; HRMS (ESI) m/z calcd. for C_12_H_18_N_2_O_2_S_2_ [(M + H)^+^] 287.0882, found 287.0880.

**[5-(1,2-dithiolan-3-yl)-N-(3-phenylisoxazol-5-yl)pentanamide] (13):** *R/S*-LA (500 mg, 2.4 mmol) was refluxed with 5-amino-3-phenylisoxazole (465 mg, 2.9 mmol) in THF (10 mL) at 66 ℃ for 24 h with CDI (589 mg, 3.6 mmol) as the coupling reagent. The desired product was isolated, using procedure “B”, as a yellowish solid (98 mg), yield: 12%; mp: 90−92 ℃; ^1^HNMR (300 MHz, CDCl_3_): δ 8.48 (s, 1H, NH Ar), δ 7.79–7.68 (m, 2H, 2CH Ar), δ 7.38 (m, 3H, 3CH Ar), δ 6.67 (s, 1H, CH Ar), δ 3.57–3.44 (m, 1H, CH), δ 3.18–2.99 (m, 2H, CH_2_), δ 2.47–2.31 (m, 3H, CH CH_2_), δ 1.91–1.77 (m, 1H, CH), δ 1.76–1.55 (m, 4H, 2CH_2_), δ 1.54–1.34 (m, 2H, CH_2_); ^13^CNMR, *δ* (ppm) (300 MHz, CDCl_3_): 169.2245, 163.8211, 160.6310, 130.2767, 128.9544, 126.8079, 87.1703, 56.3200, 40.2685, 38.5295, 36.4398, 34.5836, 28.7406, 24.7977; HRMS (ESI) m/z calcd. for C_17_H_20_N_2_O_2_S_2_ [(M + H)^+^] 349.1039, found 349.1033.

**[N'-(5-(1,2-dithiolan-3-yl)pentanoyl)-4-chlorobenzohydrazide] (14)** [28]**:** *R/S*-LA (500 mg, 2.4 mmol) was refluxed with 4-chlorobenzhydrazide (496 mg, 2.9 mmol) in THF (10 mL) at 66℃ for 24 h, using CDI (589 mg, 3.6 mmol) as the coupling reagent. The desired product was isolated, using procedure “B”, as a yellowish solid (340 mg), yield: 40%; mp: 182−185 ℃; ^1^HNMR: *δ* (ppm) (300 MHz, DMSO -d_6_) δ 10.40 (s, 1H, NH), δ 9.90 (s, 1H, NH), δ 7.88 (d, J = 8.53 Hz, 2H, 2CH Ar), δ 7.57 (d, J = 8.53 Hz, 2H, 2CH Ar), δ 3.69–3.54 (m, 1H, CH), δ 3.27–3.07 (m, 2H, CH_2_), δ 2.49–2.35 (m, 1H, CH), δ 2.20 (t, J = 7.11 Hz, 2H, CH_2_), δ 1.97–1.81 (m, 1H, CH), δ 1.78–1.50 (m, 4H, 2CH_2_), δ 1.49–1.31 (m, 2H, CH_2_); ^13^CNMR(300 MHz, DMSO-d_6_): 171.8851, 164.9207, 137.0721, 131.7220, 129.8137, 129.0569, 56.6141, 40.3891, 38.5659, 34.5810, 33.5649, 28.6978, 25.3074; HRMS (ESI) m/z calcd. for C_15_H_19_ClN_2_O_2_S_2_ [(M + H)^+^] 359.0649, found 359.0643.

**[N-(2,5-dioxopyrrolidin-3-yl)-5-(1,2-dithiolan-3-yl)pentanamide](15)**: *R/S*-LA (500 mg, 2.4 mmol) was reacted with 1-aminohydantoin hydrochloride (440 mg, 2.9 mmol) and DIPEA (1mL, 5.4 mmol) in DMF (10 mL) at room temperature for 24 h. HATU (1.1 g, 2.9 mmol) was the coupling reagent. The desired product was isolated, using procedure “B”, as a yellowish solid (120 mg), yield: 16%; mp: 148−151 ℃; ^1^HNMR(300 MHz, DMSO-d_6_): δ 11.21 (s, 1H, NH), δ 10.21 (s, 1H, NH), δ 4.02 (s, 2H, CH_2_), δ 4.02 (s, 2H, CH_2_), δ 3.70–3.54 (m, 1H, CH), δ 3.25–3.05 (m, 2H, CH_2_), δ 2.48–2.33(s, 2H, CH_2_), δ 2.14 (t, J = 7.15 Hz, 2H, CH_2_), δ 1.94–1.81 (m, 1H, CH), δ 1.74–1.47 (m, 4H, 2CH_2_), δ 1.45–1.30 (m, 2H, CH_2_); ^13^CNMR (300 MHz, DMSO-d_6_): 172.3385, 170.5409, 157.8951, 56.5259, 53.1820, 40.3376, 38.5679, 34.4834, 32.9759, 28.5078, 24.9223; HRMS (ESI) m/z calcd. for C_11_H_17_N_3_O_3_S_2_ [(M + Na)^+^] 326.0604, found 326.0604.

**[5-(1,2-dithiolan-3-yl)-N-(5-(4-fluorophenyl)-1,3,4-thiadiazol-2-yl)pentanamide] (16):** *R/S*-LA (300 mg, 1.5 mmol) was reacted with 2-amino-5-(4-fluorophenyl)-1,3,4-thiadiazole (350 mg, 1.8 mmol) and DIPEA (600 µL, 3.4 mmol) in DMF (10 mL) at room temperature for 24 h with HATU (650 mg, 1.7 mmol) as the coupling reagent. The desired product was isolated, using procedure “B”, as a yellowish solid (152 mg), yield: 27%; mp: 215−218 ℃; ^1^HNMR(300 MHz, DMSO-d_6_): δ 12.65 (s, 1H, NH), δ 8.00 (m, 2H, 2CH Ar), 7.37 (m, 2H, 2CH Ar), δ 3.72–3.55 (m, 1H, CH), δ 3.25–3.05 (m, 2H, CH_2_), δ 2.48–2.34 (m, 1H, CH), δ 1.96–1.80 (sex, J = 6.6390 Hz, 1H, CH), δ 1.78–1.50 (m, 4H, 2CH_2_), δ 1.48–1.30 (m, 2H, CH_2_); ^13^CNMR(300 MHz, DMSO-d_6_): 171.9448, 161.0643, 158.8594, 129.7432, 129.6270, 117.0473, 116.7547, 56.4645, 40.3704, 38.5985, 35.1675, 34.5119, 28.6078, 24.8054; HRMS (ESI) m/z calcd. for C_16_H_18_FN_3_OS_3_ [(M + H)^+^] 384.0669, found 384.0663.

**[N-(1-benzylpiperidin-4-yl)-5-(1,2-dithiolan-3-yl)pentanamide] (17)** [29]**:** *R/S*-LA (260 mg, 1.3 mmol) was reacted with 1-benzylpiperidine-4-amine (270 µL, 1.3 mmol) and DIPEA (500 µL, 5.2 mmol) in DMF (10 mL) at room temperature for 24 h with HATU (608 mg, 1.6 mmol) as the coupling reagent. The desired product was isolated, using procedure “B”, as a yellowish solid (68 mg), yield: 15%; mp: 79−82 ℃; ^1^HNMR (300 MHz, CDCl_3_): δ 7.36–7.21 (m, 5H, 5CH Ar), δ 5.32 (m, 1H, NH), 3.89–3.72 (m, 1H, CH), δ 3.63–3.52 (m, 1H, CH), δ 3.51–3.46 (s, 2H, CH_2_), δ 3.23–3.06 (m, 2H, CH_2_), δ 2.89–2.77 (m, 3H, CH_2_), δ 2.58–2.40 (sex, J = 6.2172 Hz, 1H, CH), δ 2.22–2.01 (m, 4H, 2CH_2_), δ 2.00–1.80 (m, 3H, CH_2_CH), δ 1.79–1.57 (m, 4H, 2CH_2_), δ 1.56–1.34 (m, 4H, 2CH_2_); ^13^CNMR(300 MHz, CDCl_3_): 171.9992, 138.1433, 129.1637, 128.2664, 127.1346, 63.0485, 56.4401, 52.2850, 46.4350, 40.2555, 38.4824, 36.6530, 34.6285, 32.2757, 28.8633, 25.4383; HRMS (ESI) m/z calcd. for C_20_H_30_N_2_OS_2_ [(M + H)^+^] 379.1872, found 379.1872.

**[5-(1,2-dithiolan-3-yl)-N-(9H-fluoren-9-yl)pentanamide] (18):** *R/S*-LA (300 mg, 1.5 mmol) was reacted with 9-Aminofluorene hydrochloride (380 mg, 1.8 mmol) and DIPEA (700 µL, 4.0 mmol) in DMF (10 mL) at room temperature for 24 h with HATU (718 mg, 1.9 mmol) as the coupling reagent. The desired product was isolated, using procedure “B”, as a yellowish solid (316 mg), yield: 59%; mp: 188−191 ℃; 1HNMR(300 MHz, DMSO-d6): δ 8.45 (d, J = 8.3447 Hz, 1H, NH), δ 7.87 (d, J = 7.3360 Hz, 2H, 2CH Ar), δ 7.53–7.38 (m, 4H, 4CH Ar), δ 7.37–7.28 (m, 2H, 2CH Ar), δ 6.04 (d, J = 8.5280 Hz, 1H, CH), δ 3.70–3.58 (m, 1H, CH), δ 3.25–3.08 (m, 2H, CH_2_), δ 2.49–2.37 (m, 1H, CH), δ 2.22 (t, J = 7.1067 Hz, 2H, CH_2_), δ 1.94–1.81 (m, 1H, CH), δ 1.77–1.34 (m, 6H, 3CH_2_); ^13^CNMR(300 MHz, DMSO-d_6_): 173.4798, 145.4370, 140.4961, 128.7982, 128.0911, 125.2703, 120.6140, 56.6285, 54.4179, 40.3966, 38.6180, 35.5524, 34.5898, 28.6706, 25.6814; HRMS-(ESI) m/z calcd. for C_21_H_23_NOS_2_ [(M + H)^+^] 370.1294, found 370.1292.

**[5-(1,2-dithiolan-3-yl)-N-(furan-3-ylmethyl)pentanamide] (19):** *R/S*-LA (250 mg, 1.2 mmol) was reacted with 3-furylmethylamine (130 mg, 1.3 mmol) and DIPEA (700 µL, 4 mmol) in DMF (10 mL) at room temperature for 24 h with HATU (605 mg, 1.6 mmol) as the coupling reagent. The desired product was isolated, using procedure “B”, as a yellowish solid (100 mg), yield: 24%; mp: 52−54 ℃; 1HNMR(300 MHz, DMSO-d6): δ 8.14 (m, 1H, NH), δ 7.59 (m, 1H, CH Ar), δ 7.52 (m, 1H, CH Ar), δ 6.40 (s, 1H, CH Ar), δ 4.08 (d, J = 5.5937 Hz, 2H, CH_2_), δ 3.66–3.55 (m, 1H, CH), δ 3.24–3.07 (m, 2H, CH_2_), δ 2.47–2.34 (m, 1H, CH), δ 2.10 (t, J = 7.2901 Hz, 2H, CH_2_), δ 1.92–1.79 (m, 1H, CH) δ 1.76–1.44 (m, 4H, CH_2_), δ 1.42–1.24 (m, 2H, CH_2_); ^13^CNMR(300 MHz, DMSO-d_6_):172.3005, 143.7706, 140.1421, 123.8400, 111.0567, 56.5924, 40.3702, 38.5716, 35.5521, 34.5878, 33.6794, 28.7855, 25.4973; HRMS (ESI) m/z calcd. for C_13_H_19_NO_2_S_2_ [(M + H)^+^] 286.0930, found 286.0929.

**[N-((2,3-dihydro-1H-inden-1-yl)methyl)-5-(1,2-dithiolan-3-yl)pentanamide] (20):** *R/S*-LA (263 mg, 1.3 mmol) was reacted with 1-(2,3-dihydro-1H-inden-1-yl)methanamine (248 mg, 1.4 mmol) and DIPEA (700 µL, 4 mmol) in DMF (10 mL) at room temperature for 24 h with HATU (570 mg, 1.5 mmol) as the coupling reagent. The desired product was isolated, using procedure “B”, as a yellowish solid (56 mg), yield: 14%; mp: 115−118 ℃; 1HNMR(300 MHz, DMSO-d6): δ 7.30–7.12 (m, 4H, 4CH Ar), δ 5.54 (s, 1H, NH), 3.72–3.50 (m, 2H, CH_2_), δ 3.48–3.31 (m, 2H, CH_2_), δ 3.24–3.06 (m, 2H, CH_2_), δ 3.02–2.80 (m, 2H, CH_2_), δ 2.52–2.40 (m, 1H, CH), δ 2.33–2.21 (m, 1H, CH), δ 2.20–2.12 (m, 2H, CH_2_), δ 1.97–1.74 (m, 1H, CH), δ 1.73–1.55 (m, 5H, 2CH_2_ CH), δ 1.54–1.34 (m, 2H, CH_2_); ^13^CNMR(300 MHz, CDCl_3_): 172.9120, 144.4857, 143.9918, 127.0698, 126.3863, 124.8711, 123.7653, 56.4331, 44.7112, 42.7514, 40.2643, 38.5012, 36.5770, 34.6581, 31.2452, 29.5841, 28.9103, 25.4380; HRMS (ESI) m/z calcd. for C_18_H_25_NOS_2_ [(M + H)^+^] 336.1450, found 336.1449.

**[1-(4-(9H-fluoren-9-yl)piperazin-1-yl)-5-(1,2-dithiolan-3-yl)pentan-1-one] (21):** *R/S*-LA (334 mg, 1.6 mmol) was reacted with 1-(9H-fluoren-9-yl)piperazine (483 mg, 1.9 mmol) and DIPEA (700 µL, 4 mmol) in DMF (10 mL) at room temperature for 24 h with HATU (740 mg, 1.9 mmol) as the coupling reagent. The desired product was isolated, using procedure “B”, as a yellowish solid (525 mg), yield: 83%; mp: 119−122 ℃; 1HNMR(300 MHz, DMSO-d6): δ 7.69 (d, J = 7.4277 Hz, 1H, 2CH Ar), δ 7.60 (d, J = 7.3360 Hz, 2H, 2CH Ar), δ 7.38 (t, J = 7.2443 Hz, 1H, 2CH Ar), δ 7.33–7.25 (m, 2H, 2CH Ar), δ 4.86 (s, 1H, CH), δ 3.67–3.58 (m, 2H, CH_2_), δ 3.57–3.50 (m, 1H, CH), δ 3.41 (m, 2H, CH_2_), δ 3.22–3.04 (m, 2H, CH_2_), δ 2.73 (m, 2H, CH_2_), δ 2.51–2.38 (m, 3H, CH_2_ CH), δ 2.26 (t, J = 7.3818 Hz, 2H, CH_2_), δ 1.96–1.82 (m, 1H, CH), δ 1.75–1.53 (m, 4H, 2CH_2_), δ 1.53–1.34 (m, 2H, CH_2_); ^13^CNMR(300 MHz, CDCl_3_): 171.1196, 143.4397, 141.0402, 128.3107, 127.1544, 125.9073, 119.8402, 69.9317, 56.4537, 49.6125, 48.4564, 46.2708, 42.3715, 40.2410, 38.6320, 38.4939, 34.7594, 32.9358, 29.1109, 24.9850; HRMS (ESI) m/z calcd. for C_25_H_30_N_2_OS_2_ [(M + H)^+^] 439.1872, found 439.1871.

## Supporting information

S1 File SwissADME drug likeness data for all 21 compounds. (DOCX)

S2 File Individual ^1^H/^13^C NMR spectra for 21 compounds. (DOCX)

S3 File HRMS data. (DOCX)
