# Supplementary material for: Bis-hydrophobic 5-(1,2-dithiolan-3-yl)pentanamide budding leads targeting brain sigma-1 receptors
Source: PLoS One. 2026 Aug 10;21(8):e0352906. doi: 10.1371/journal.pone.0352906 (PMC13456256; doi:10.1371/journal.pone.0352906)
Supplement: Supplementary file 1 [file pone.0352906.s001.docx]

Supplemental 1 File: SwissADME Data set

Compound 1

Compound 2

Compound 3

Compound 4

Compound 5

Compound 6

Compound 7

Compound 8

Compound 9

Compound 10

Compound 11

Compound 12

Compound 13

Compound 14

Compound 15

Compound 16

Compound 17

Compound 18

Compound 19

Compound 20

Compound 21
