# Supplementary material for: Bis-hydrophobic 5-(1,2-dithiolan-3-yl)pentanamide budding leads targeting brain sigma-1 receptors
Source: PLoS One. 2026 Aug 10;21(8):e0352906. doi: 10.1371/journal.pone.0352906 (PMC13456256; doi:10.1371/journal.pone.0352906)
Supplement: Supplementary file 2 [file pone.0352906.s002.docx]

**Supplemental File 2: 1H/13C Nuclear Magnetic Resonance (NMR) Spectra Data**

**Compound 1:** Known [22]

HNMR:

CNMR:

**Compound 2:** Known [23].

HNMR:

CNMR:

**Compound 3:** Known [24].

HNMR:

CNMR:

**Compound 4:** Known [25].

HNMR:

CNMR:

**Compound 5:** Unknown.

HNMR:

CNMR:

**Compound 6:** Known [26].

HNMR:

CNMR:

**Compound 7:** Known [27].

HNMR:

CNMR:

**Compound 8:** Unknown.

HNMR:

CNMR:

**Compound 9:** Unknown.

HNMR:

CNMR:

**Compound 10:** Unknown.

HNMR:

CNMR:

**Compound 11:** Unknown.

HNMR:

CNMR:

**Compound 12:** Unknown.

CNMR:

CNMR:

**Compound 13:** Unknown.

HNMR:

CNMR:

**Compound 14:** Known [28].

HNMR:

CNMR:

**Compound 15:** Unknown.

HNMR:

CNMR:

**Compound 16:** Unknown.

HNMR:

CNMR:

**Compound 17**: Known [29].

HNMR:

CNMR:

**Compound 18:** Unknown

HNMR:

CNMR:

**Compound 19:** Unknown.

HNMR:

CNMR:

**Compound 20:** Unknown.

HNMR:

CNMR:

**Compound 21:** Unknown.

HNMR:

CNMR:
