# Supplementary material for: Bis-hydrophobic 5-(1,2-dithiolan-3-yl)pentanamide budding leads targeting brain sigma-1 receptors
Source: PLoS One. 2026 Aug 10;21(8):e0352906. doi: 10.1371/journal.pone.0352906 (PMC13456256; doi:10.1371/journal.pone.0352906)
Supplement: Supplementary file 3 [file pone.0352906.s003.docx]

Supplemental File 3: High Resolution Mass Spectrometry (HRMS) Data

| **Compounds** | Mol. Formula | Adduct | *m/z* value Theoretical | *m/z* value Experimental | Mass accuracy in ppm | Structure |
| --- | --- | --- | --- | --- | --- | --- |
|  |  | (H^+^, Na^+^, Cs^+^) |  |  |  |  |
| **1** | C17H23NO3S2 | [M+H^+^]^+^ | 354.1192 | 354.1190 | 0.56 |  |
| **2** | C13H21N3OS2 | [M+H^+^]^+^ | 300.1199 | 300.1196 | 1.00 |  |
| **3** | C16H21NO3S2 | [M+H^+^]^+^ | 340.1036 | 340.1032 | 1.18 |  |
| **4** | C15H22N2OS2 | [M+H^+^]^+^ | 311.1246 | 311.1243 | 0.96 |  |
| **5** | C15H18N2O2S2 | [M+H^+^]^+^ | 323.0882 | 323.0877 | 1.55 |  |
| **6** | C15H19N3OS2 | [M+H^+^]^+^ | 322.1042 | 322.1037 | 1.55 |  |
| **7** | C15H22N2O3S3 | [M+H^+^]^+^ | 375.0865 | 375.0858 | 1.87 |  |
| **8** | C13H20N2OS3 | [M+H^+^]^+^ | 317.0811 | 317.0807 | 1.26  |  |
| **9** | C11H19N5OS2 | [M+H^+^]^+^ | 302.1104 | 302.1103 | 0.33 |  |
| **10** | C16H24N4OS2 | [M+H^+^]^+^ | 353.1464 | 353.1457 | 1.98 |  |
| **11** | C17H20N2OS2 | [M+H^+^]^+^ | 333.1090 | 333.1085 | 1.50 |  |
| **12** | C12H18N2O2S2 | [M+H^+^]^+^ | 287.0882 | 287.0880 | 0.70  |  |
| **13** | C17H20N2O2S2 | [M+H^+^]^+^ | 349.1039 | 349.1033 | 1.72 |  |
| **14** | C15H19ClN2O2S2 | [M+H^+^]^+^ | 359.0649 | 359.0643 | 1.67 |  |
| **15** | C11H17N3O3S2 | [M+Na^+^]^+^ | 326.0604 | 326.0604 | 0.00 |  |
| **16** | C16H18FN3OS3 | [M+H^+^]^+^ | 384.0669 | 384.0663 | 1.56 |  |
| **17** | C20H30N2OS2 | [M+H^+^]^+^ | 379.1872 | 379.1872 | 0.00 |  |
| **18** | C21H23NOS2 | [M+H^+^]^+^ | 370.1294 | 370.1292 | 0.54 |  |
| **19** | C13H19NO2S2 | [M+H^+^]^+^ | 286.0930 | 286.0929 | 0.35 |  |
| **20** | C18H25NOS2 | [M+H^+^]^+^ | 336.1450 | 336.1449 | 0.30 |  |

| **21** | C25H30N2OS2 | [M+H^+^]^+^ | 439.1872 | 439.1871 | 0.23 |  |
| --- | --- | --- | --- | --- | --- | --- |

Method	tune_pos_low_DI-APCI.m	Operator

Sample Name Comment

# Compd - 01

## 95/4.9/0.1% ACN/H2O/FA

ESI positive

Instrument	maXis plus

Method	tune_pos_low_DI-APCI.m	Operator

Sample Name Comment

# Compd - 02

## 95/4.9/0.1% ACN/H2O/FA

ESI positive

Instrument	maXis plus

Method	tune_pos_low_DI-APCI.m	Operator

Sample Name Comment

# Compd - 03

## 95/4.9/0.1% ACN/H2O/FA

ESI positive

Instrument	maXis plus

Method	tune_pos_low_DI-APCI.m	Operator

Sample Name Comment

# Compd - 04

## 95/4.9/0.1% ACN/H2O/FA

ESI positive

Instrument	maXis plus

Method	tune_pos_low_DI-APCI.m	Operator

Sample Name Comment

# Compd - 05

## 95/4.9/0.1% ACN/H2O/FA

ESI positive

Instrument	maXis plus

Method	tune_pos_low_DI-APCI.m	Operator

Sample Name Comment

# Compd - 06

## 95/4.9/0.1% ACN/H2O/FA

ESI positive

Instrument	maXis plus

Method	tune_pos_low_DI-APCI.m	Operator

Sample Name Comment

# Compd - 07

## 95/4.9/0.1% ACN/H2O/FA

ESI positive

Instrument	maXis plus

Method	tune_pos_low_DI-APCI.m	Operator

Sample Name Comment

# Compd - 08

## 95/4.9/0.1% ACN/H2O/FA

ESI positive

Instrument	maXis plus

Method	tune_pos_low_DI-APCI.m	Operator

Sample Name Comment

# Compd - 09

## 95/4.9/0.1% ACN/H2O/FA

ESI positive

Instrument	maXis plus

Method	tune_pos_low_DI-APCI.m	Operator

Sample Name Comment

# Compd - 10

## 95/4.9/0.1% ACN/H2O/FA

ESI positive

Instrument	maXis plus

Method	tune_pos_low_DI-APCI.m	Operator

Sample Name Comment

# Compd - 11

## 95/4.9/0.1% ACN/H2O/FA

ESI positive

Instrument	maXis plus

Method	tune_pos_low_DI-APCI.m	Operator

Sample Name Comment

# Compd - 12

## 95/4.9/0.1% ACN/H2O/FA

ESI positive

Instrument	maXis plus

Method	tune_pos_low_DI-APCI.m	Operator

Sample Name Comment

# Compd - 13

## 95/4.9/0.1% ACN/H2O/FA

ESI positive

Instrument	maXis plus

Method	tune_pos_low_DI-APCI.m	Operator

Sample Name Comment

# Compd - 14

## 95/4.9/0.1% ACN/H2O/FA

ESI positive

Instrument	maXis plus

Method	tune_pos_low_DI-APCI.m	Operator

Sample Name Comment

# Compd - 15

## 95/4.9/0.1% ACN/H2O/FA

ESI positive

Instrument	maXis plus

Method	tune_pos_low_DI-APCI.m	Operator

Sample Name Comment

# Compd - 16

## 95/4.9/0.1% ACN/H2O/FA

ESI positive

Instrument	maXis plus

Method	tune_pos_low_DI-APCI.m	Operator

Sample Name Comment

# Compd - 17

## 95/4.9/0.1% ACN/H2O/FA

ESI positive

Instrument	maXis plus

Method	tune_pos_low_DI-APCI.m	Operator

Sample Name Comment

# Compd - 18

## 95/4.9/0.1% ACN/H2O/FA

ESI positive

Instrument	maXis plus

Method	tune_pos_low_DI-APCI.m	Operator

Sample Name Comment

# Compd - 19

## 95/4.9/0.1% ACN/H2O/FA

ESI positive

Instrument	maXis plus

Method	tune_pos_low_DI-APCI.m	Operator

Sample Name Comment

# Compd - 20

## 95/4.9/0.1% ACN/H2O/FA

ESI positive

Instrument	maXis plus

Method	tune_pos_low_DI-APCI.m	Operator

Sample Name Comment

# Compd - 21

## 95/4.9/0.1% ACN/H2O/FA

ESI positive

Instrument	maXis plus
